# Microwave assisted synthesis of Mn_3_O_4_ nanograins intercalated into reduced graphene oxide layers as cathode material for alternative clean power generation energy device

**DOI:** 10.1038/s41598-022-23622-x

**Published:** 2022-11-09

**Authors:** Mehmood Shahid, Thilina Rajeendre Katugampalage, Mohammad Khalid, Waqar Ahmed, Chariya Kaewsaneha, Paiboon Sreearunothai, Pakorn Opaprakasit

**Affiliations:** 1 School of Integrated Science and Innovation (ISI), Sirindhorn International Institute of Technology (SIIT), Thammasat University, Rangsit, 12121 Pathum Thani Thailand; 2 Graphene and Advanced 2D Materials Research Group (GAMRG), School of Engineering and Technology, Sunway University, No. 5, Jalan Universiti, Bandar Sunway, 47500 Subang Jaya, Selangor Malaysia; 3 Malaysia – Japan International Institute of Technology (MJIIT), Universiti Teknologi Malaysia, Jalan Sultan Yahya Petra, 54100 Kuala Lumpur, Malaysia

**Keywords:** Fuel cells, Renewable energy, Materials science

## Abstract

Mn_3_O_4_ nanograins incorporated into reduced graphene oxide as a nanocomposite electrocatalyst have been synthesized via one-step, facile, and single-pot microwave-assisted hydrothermal technique. The nanocomposites were employed as cathode material of fuel cells for oxygen reduction reaction (ORR). The synthesized product was thoroughly studied by using important characterization, such as XRD for the structure analysis and FESEM and TEM analyses to assess the morphological structures of the material. Raman spectra were employed to study the GO, rGO bands and formation of Mn_3_O_4_@rGO nanocomposite. FTIR and UV–Vis spectroscopic analysis were used to verify the effective synthesis of the desired electrocatalyst. The Mn_3_O_4_@rGO-10% nanocomposite with 10 wt% of graphene oxide was used to alter the shiny surface of the working electrode and applied for ORR in O_2_ purged 0.5 M KOH electrolyte solution. The Mn_3_O_4_@rGO-10% nanocomposite electrocatalyst exhibited outstanding performance with an improved current of − 0.738 mA/cm^2^ and shifted overpotential values of − 0.345 V when compared to other controlled electrodes, including the conventionally used Pt/C catalyst generally used for ORR activity. The tolerance of Mn_3_O_4_@rGO-10% nanocomposite was tested by injecting a higher concentration of methanol, i.e., 0.5 M, and found unsusceptible by methanol crossover. The stability test of the synthesized electrocatalyst after 3000 s was also considered, and it demonstrated excellent current retention of 98% compared to commercially available Pt/C electrocatalyst. The synthesized nanocomposite material could be regarded as an effective and Pt-free electrocatalyst for practical ORR that meets the requirement of low cost, facile fabrication, and adequate stability.

## Introduction

Day-by-day growing needs, the rapid development of the global economy, and technology have led to the diminution of fossil fuel reserves, resulting in an energy crisis and global warming issues^1^. Although present energy demands are being met by conventional fossil fuel reserves, these energy sources need to be reserved for future generations^2^. This looming energy crisis has driven researchers to look for sustainable, cost-effective, environmentally friendly, and efficient alternative energy sources^3^. Therefore, in search of alternative energy sources, a tremendous amount of effort has been made to find renewable energy sources. Li-Ion batteries, supercapacitors, fuel cells, and solar cells as electrochemical energy storage/conversion devices have gained considerable attention^4–8^. Rechargeable metal-air batteries (MABs) and proton exchange membrane fuel cells (PEMFCs) are next-generation energy sources for producing clean electricity^9,10^. In these devices, O_2_ reduction takes place at the cathode surface. In energy conversion devices (fuel cells), the O_2_ reduction reaction is a critical process. Oxygen reduction reaction (ORR) happens in two main pathways in an aqueous solution; (1) a four-electron transition that reduces O_2_ to H_2_O (water) and (2) a two-electron transfer mechanism where O_2_ is reduced to H_2_O_2_ (Hydrogen peroxide). In the case of aprotic non-aqueous solvents or alkaline solutions, a 1 electron reduction phenomenon can also occur by reducing O_2_ into superoxide (O_2_^−^). In the case of proton exchange membrane (PEM) fuel cell operation, the O_2_ molecules are reduced at the cathode surface by gaining electrons due to ORR. The O=O bond with an exceptionally strong bonding energy of 489 kJ/mol^11^ is a powerful bond that is very difficult to break electrochemically. To reduce this energy barrier and the bond activation and cleavage, the assistance of electrocatalysts is highly required.

ORR is a six-time slower process at the cathode surface than that of hydrogen oxidation in an aqueous solution in PEMFCs. This slow O_2_ reduction arises due to the varied reaction pathways and adsorption/desorption process due to the participation of O-containing intermediate species such as OOH^*^, O^*^, and OH^*^^12^. Because of this reason, the cathode catalyst requirement is often ten times higher than the anode catalyst requirement for fuel cell applications^13^. On an industrial scale, conventionally-used Pt-based electrocatalyst for ORR accounts for 36–56% of the total cost of fuel cells^14,15^. Although the high-cost Pt-based electrocatalyst is a significant concern, another drawback of conventionally-used electrocatalyst is its susceptibility to fuel crossover, due to which the stability of the fuel cell is compromised, hence tremendously limiting the fuel cells’ massive applications. Consequently, creating a highly active, sufficiently stable, and economical electrocatalyst is of supreme importance to replace the Pt-based cathode electrode for large-scale applications.

To develop a cathode of fuel cells that is economical, abundantly available, and has higher performance as an alternative to Pt-based catalyst, researchers have proposed various metal oxides^16–18^, metal sulfides^19,20^, metal-based materials^21,22^ in the form of the unary, binary and ternary nanocomposite. It was further discovered that combining the above-discussed cathode material with carbon support can further enhance their activity for ORR because of the supporting material's higher surface area^23^. In this work, we have developed metal oxide supported on various concentrations of carbon matrix, *i.e*., reduced graphene oxide (rGO) layers, and exploited for ORR. The synthesized cathode catalyst has rivalled the conventionally used Pt-based catalyst for ORR. This report presents the synthesis of Mn_3_O_4_ nanoparticles incorporated into various concentrations (wt%) of GO (5, 10, and 15 wt%) w.r.t. Mn_3_O_4_ precursor using the microwave hydrothermal technique.

The microwave hydrothermal technique is a novel powder preparation process that has emerged in recent years. It employs microwaves for heating and operates on the hydrothermal principle; however, it differs from the typical hydrothermal synthesis process. The microwave hydrothermal technology combines hydrothermal and microwave technologies, maximizing the benefits of microwaves and water heating. In contrast to the hydrothermal method, the microwave hydrothermal heating method employs microwaves rather than a single conduction approach. Even if the sample has a certain depth, microwaves may enter it and heat each depth simultaneously, eliminating heat conduction, resulting in a temperature differential, and considerably boosting reaction speed. In comparison to the traditional hydrothermal method, the microwave hydrothermal method has a faster heating speed, a more sensitive reaction, and a more uniform heating system, allowing it to swiftly generate nanoparticles with a consistent particle size distribution and shape^24^.

A microwave-assisted hydrothermal method is low-cost and eco-friendly, with low environmental impacts and little processing time. The primary advantage of this synthesis process is its efficient energy transfer and fast volumetric heating^25^ compared to the ordinary hydrothermal methods generally carried out in the conventional heating oven, which cost longer synthesis time and slow process. The microwave-assisted hydrothermal method is a low-energy consumption method with high yield and selective heating^26^. Furthermore, the nanocomposite synthesized via microwave-assisted hydrothermal method consists of multiple characteristics that include a high yield, facile preparation method, single step, and low energy consumption^27–30^.

Graphene-based nanocomposite synthesized by microwave-assisted hydrothermal process has attracted intensive attention nowadays from researchers for various applications^31^. The use of microwaves in hydrothermal synthesis has great applicability when making carbon-based material due to their microwave absorbance. Using the microwave-assisted synthesis for graphene-based nanocomposite can promote various reactions, including synthesis of desired material/nanocomposite, reduction and exfoliation of graphene, doping, wrapping, and decoration of metal/metal oxides to the graphene surface^32^. The novel composite electrode material synthesized by microwaves assisted method based on graphene derivatives containing metals/metal oxides has shown great applicability and improved performance for electrochemical applications. This method of synthesis helps the metal/metal oxide material to be anchored, intercalated, and wrapped into various layers of carbon materials (rGO in this case), which eventually promotes faster electron transfer, higher surface area and also allows the electrolyte to interact and diffuses into the sample layers^33^. The slight changes in microwave power, reaction time and variation of solvent and additives can help in the synthesis of material with diverse morphologies and characteristics of the synthesized material^34^. Based on the facts explained, we have preferred to use the hydrothermal synthesis method for Mn_3_O_4_@rGO nanocomposites for ORR activity.

To date, numerous carbon-based metal oxide nanocomposite materials have been developed and employed for various energy related fields^35–37^, specifically for ORR^3,38,39^. Most of the reported work mainly focused on the application part by using complicated synthesis techniques which involved various steps and more chemistry^40–43^. The main goal of this project was to develop a composite material with versatile features and comparable performance for ORR as presented by the Pt/C electrocatalyst. Therefore, aiming this idea in mind, we have used a very practical, facile, reproducible, and faster synthesis technique based on the microwave-assisted hydrothermal method. Employing the microwave-assisted hydrothermal method, we have synthesized the electrocatalyst for energy application which is 36 times quicker than we reported earlier^3^. Hence the microwave-assisted hydrothermal method is a highly recommended, reliable, time and energy-saving method for various technological applications. The Mn_3_O_4_@rGO nanocomposites synthesized by using the microwave-assisted hydrothermal method have shown greater stability, low overpotential values, well defined higher O_2_ reduction peaks for fuel cell applications.

The Mn_3_O_4_@rGO nanocomposite was synthesized for ORR in an alkaline medium. Alkaline media for non-Pt-based electrocatalysts provides a suitable environment for ORR without affecting the catalyst performance, with no detrimental effects and less corrosivity^44^. The as-synthesized Mn_3_O_4_@rGO nanocomposites were characterized sufficiently by employing XRD, TEM, FESEM, EDX mapping, FTIR, BET, Raman, two probe conductivity tests, and UV–vis techniques. The electrochemical study of nanocomposite was investigated as cathode material of fuel cells for ORR, and methanol tolerance was studied by injecting a higher concentration of CH_3_OH molecules into the electrochemical cell. The stability study of the nanocomposite is also conducted in comparison with the conventionally used commercially available Pt/C electrocatalyst for ORR.

## Experimental procedure

### Materials

The chemicals and reagents purchased were of analytical quality and used as it is without any further purification. For the synthesis of desired electrode material, Graphite flakes were obtained from Asbury Graphite Inc., Rodeo (USA). The following chemicals were bought from R & M Chemicals, Selangor, Malaysia: sulfuric acid, 98%; phosphoric acid, 88%; hydrochloric acid, 35%; potassium permanganate, 99%; and ammonia solution, 25%. We bought potassium hydroxide and manganese (II) acetate tetrahydrate from Sigma Aldrich, Malaysia. Hydrogen peroxide (H_2_O_2_ 35%) and methanol (CH_3_OH) were acquired, Malaysia as well. All of the experimental work was done with DI water.

## Mn_3_O_4_@rGO nanocomposite synthesis

**Figure 1:**
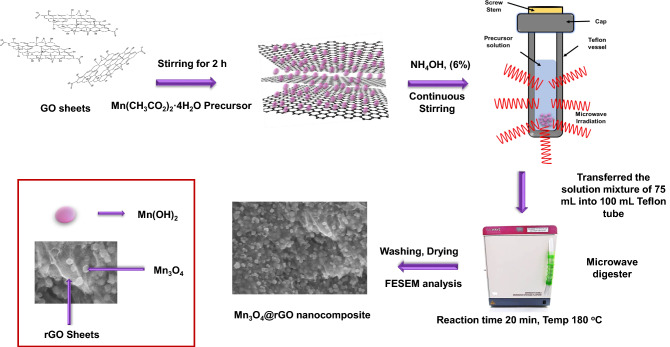
The steps of Mn_3_O_4_@rGO nanocomposites synthesis. Illustrations were generated using ChemSketch, version 2021.1. 2 and Vesta software.

The Simplified Hummer's technique was adopted for graphene oxide (GO) synthesis^45^. In brief, 3 g of graphite flakes were mixed and dissolved in H_2_SO_4_ and H_3_PO_4_ (9:1) mixture under a continuous stirring process. Afterwards, KMnO_4_ (18 g) was added to the subject solution very slowly under stirring by continuously monitoring the temperature. The solution was then left under stirring for three days with proper monitoring for complete oxidation of graphite flakes. After three days, an H_2_O_2_ solution containing ice was added to the above mixture to quench the reaction considering that 3 days were enough for oxidizing graphite. After adding H_2_O_2,_ the dark green color solution turned into a yellow color solution, indicating a higher level of graphite oxidation. The washing process was conducted, and the oxidation stopped by using 1 M HCl solution, followed by a rigorous and long washing process with DI water to remove the acid from the resulting product and reach a pH of 5 to 6. An ultra-high speed centrifugation procedure was used to wash supernatants for decantation, which also helped in the exfoliation of graphite oxide into multi-layered GO gel. The synthesized GO solution was further used for the preparation of electrode material of fuel cell with Mn_3_O_4_ as composite. The prepared GO solution was diluted to 1 mg/ml concentration and sonicated to exfoliate further the stacked layer of GO, followed by again centrifuging to collect the supernatant by leaving the multi-layer GO at the bottom. In detail, 1 mmol of Mn(CH_3_CO_2_)_2_·4H_2_O precursor prepared in 15 ml of DI water solvent was slowly added dropwise with a rate of 1 drop/sec into the different wt% (5, 10, 15 wt%) of GO solution under a continuous stirring process. Slowly adding Mn(CH_3_CO_2_)_2_·4H_2_O precursor solution into GO provides sufficient time for Mn ions to bond electrostatically with GO functional groups to make nanocomposites. The prepared solution was kept under stirring for 2 h to give the mixture a maximum time so that the reaction between the precursor solution and GO could happen. After that, a low concentration of ammonia (6%) solution was dropwise added to the mixture to achieve a basic pH (of 10). The ammonia solution will support the conversion of GO to rGO and the precipitation of manganese ions^46–48^. The Teflon tubes of 100 ml was filled with the prepared mixture up to 75%, appropriately sealed and placed into a microwave digester for hydrothermal reaction. The mixture was kept under reaction conditions for about 20 min at 180 °C. After the completion of reaction, the synthesized product was allowed to cool down, and the precipitates were then collected, washed with DI water and ethanol, and adequately packed. After the washing process, the product was dried, crushed, and stored for further analysis. The exact process was repeated for the rest of the nanocomposites by varying the GO wt%. The same experimental procedures were also used to create the control samples. The prepared nanocomposites were named Mn_3_O_4_@rGO-5%, Mn_3_O_4_@rGO-10%, and Mn_3_O_4_@rGO-15%, respectively. The schematic in Fig. 1 show the steps of Mn_3_O_4_@rGO nanocomposites synthesis.

### Characterizations

The phase identifications of as-synthesized samples were performed on a Bruker D8 advance using copper Ka radiation (λ = 1.5418 nm) under 40 kV and 40 mA with a scan rate of 0.02-degree sec^−1^. The morphological and structural properties of the prepared samples were analyzed by SEM and TEM on a Hitachi SU8030 with an acceleration voltage between 3 and 10 kV, fitted with an EXD mapping tool and JEOL :JEM2010 respectively. Raman spectra were taken using SENTERRA Dispersive Raman Microscope by Bruker with a laser excitation wavelength of 532 nm. UV–Vis and FTIR measurements were conducted on a Thermo Scientific GENESYS 180 spectrophotometer and Thermo Scientific Nicolet iS5 with a diamond crystal ATR, respectively.

### Electrode preparation for ORR and electrochemical studies

The electrochemical performance of Mn_3_O_4_@rGO nanocomposites electrode with various GO concentrations was evaluated towards ORR. A glassy carbon electrode (GCE) modified with catalyst ink was employed as a working electrode for ORR measurement. GCE's surface was physically polished using 0.05 mm alumina polishing paste before modification. Additionally, GCE was electrochemically cleaned in a 0.5 M H_2_SO_4_ solution with a potential range of − 1 to 1 V for about 100 cycles to remove any adsorbed material on the GCE surface, followed by a 5 min sonication. The mirror-like polished surface of the GCE (D = 3 mm) was dropped cast with a catalyst ink of 5 μL and 1 mg/ml concentration, and was then dried at ambient temperature for electrochemical studies.

The electrochemical studies were performed in a typical three-electrode electrochemical cell at room temperature using a Versa stat 4F potentiostat/galvanostat from Princeton Applied Research. The modified GCE served as a working electrode, whereas the reference and counter electrodes were SCE and Pt wire, respectively. A 0.5 M KOH was used as an electrolyte solution, and all electrochemical tests were performed at room temperature. The cyclic voltammetry studies were taken at the potential range from 0 to − 0.8 at a scan rate of 50 mVs^−1^. Scan rate studies, stability tests, and tolerance of electrocatalysts were also evaluated and explained in detail in the results and discussion.

## Results and discussion

### XRD analysis

**Figure 2:**
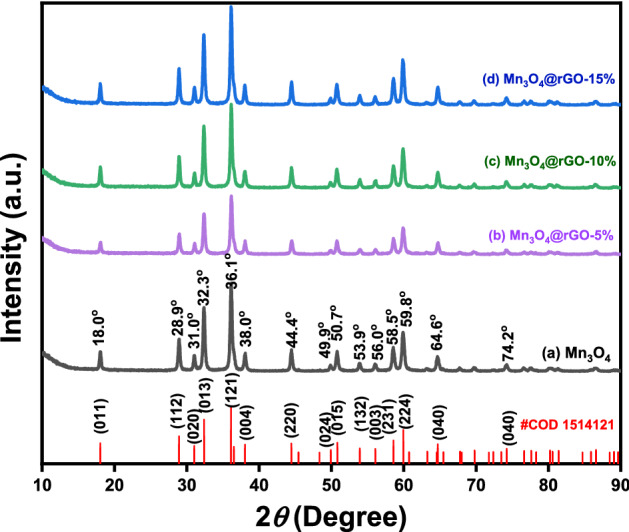
XRD patterns of (**a**) Mn_3_O_4_, (**b**) Mn_3_O_4_@rGO-5%, (**c**) Mn_3_O_4_@rGO-10%, and (**d**) Mn_3_O_4_@rGO-15%, and the standard data of Mn_3_O_4_ from COD database #1514121.

XRD patterns of Mn_3_O_4_ nanoparticles and Mn_3_O_4_@rGO nanocomposites are shown in Fig. 2. All characteristic peaks of Mn_3_O_4_ agree well with the standard data of Mn_3_O_4_ (COD #1514121) with the space group I_41_/amd^49^. No impurities were observed in the XRD patterns of both bare Mn_3_O_4_ and Mn_3_O_4_@rGO nanocomposites. Furthermore, the crystal phase of rGO was not observed, this might be due to the amorphous nature of rGO, and the smaller Mn_3_O_4_ might have completely covered the surfaces of layered rGO, which causes a low degree of graphitization.

The diffraction pattern of Mn_3_O_4_ is observable in all composites with different wt% of GO contents. Strong crystalline peaks of the pure Mn_3_O_4_ were observed at 2θ values and their corresponding crystal planes of 18.0° (011), 28.9° (112), 31.0° (020), 32.3° (013), 36.1° (121), 38.0° (044), 44.4° (220), 49.9° (024), 50.7° (015), 53.9° (132), 56.0° (033), 58.5° (231), 59.8° (224), 64.6° (040) and 74.2° (143). Correspondingly, the amount of Mn_3_O_4_ in the composite led to an increase in the diffraction peaks' intensity. Mn_3_O_4_@rGO-15% composite shows the highest intensity, while Mn_3_O_4_@rGO-5% shows the least. The Debye–Scherrer equation was used to calculate the crystallite size from the peak width is as follows^50^:

$$D = 0.9\lambda /B \cos \theta$$

where D denotes crystal size, and B is a value of FWHM of selected peaks. In order to determine the values to estimate crystal size, the Gaussian function was used to fit diffraction peaks. The average particle sizes of bare Mn_3_O_4_, Mn_3_O_4_@rGO-5%, Mn_3_O_4_@rGO-10%, and Mn_3_O_4_@rGO-15% are 33.29, 29.97, 29.10, and 29.49 nm, respectively.

### Morphological characterization of Mn_3_O_4_@rGO nanocomposites

**Figure 3:**
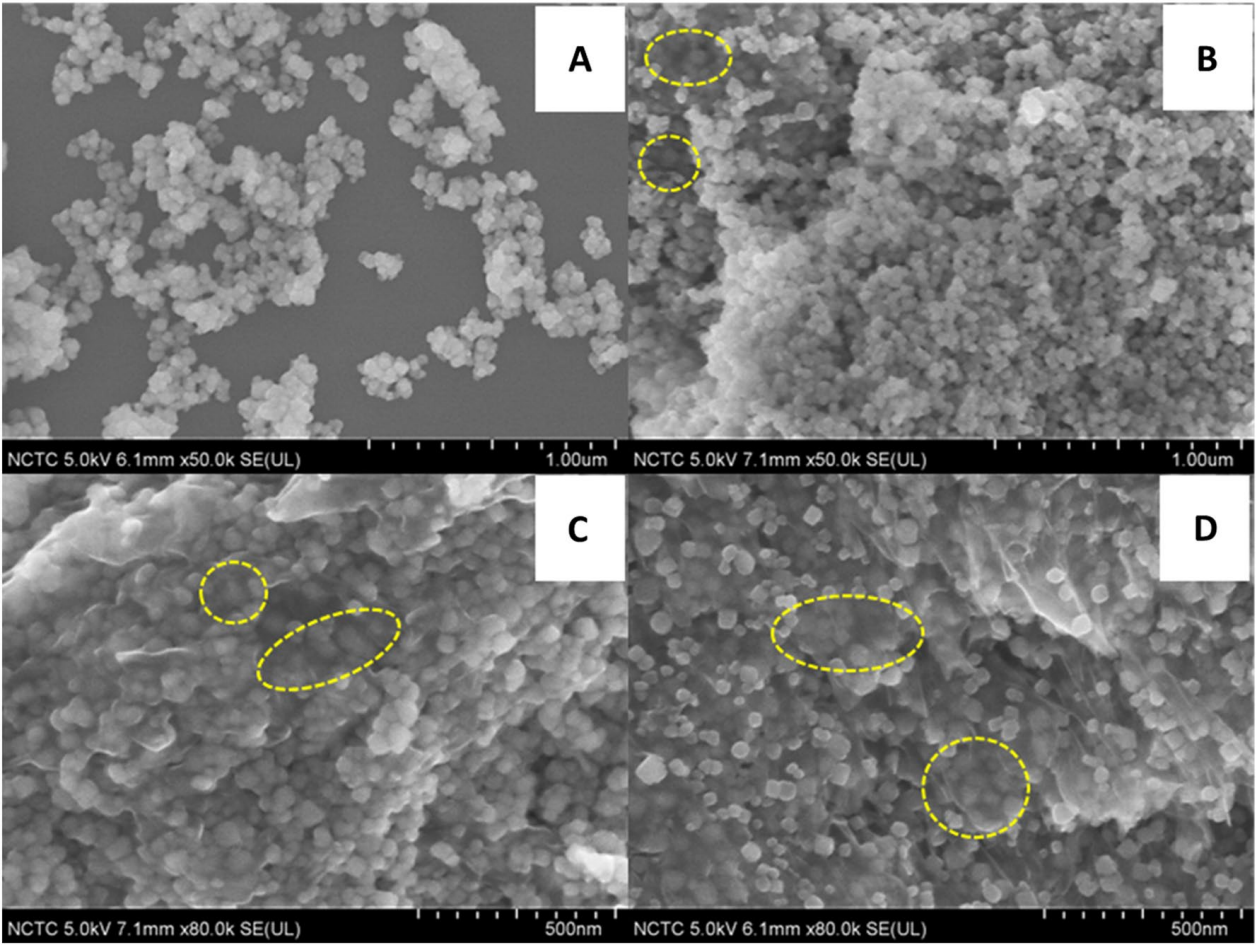
FESEM images of (**A**) Mn_3_O_4_, (**B**) Mn_3_O_4_@rGO-5%, (**C**) Mn_3_O_4_@rGO-10%, and (**D**) Mn_3_O_4_@rGO-15%.

The morphological structures of the composites were inspected using FESEM analysis techniques fitted with EDX mapping, as shown in Fig. 3. Mn_3_O_4_ nanoparticles showed granular structures. Highly aggregated Mn_3_O_4_ nanoparticles were observed as it is widely recognized that metal oxide nanoparticles precipitate in agglomerated form after a hydrothermal process. Due to the agglomerated nature of Mn_3_O_4_ nanograins-based semiconductors, the electrocatalytic reduction of O_2_ suffered from low ion transportation problems, as can be seen through the electrochemical studies in Fig. 9. The incorporation of carbon matrix in the form of rGO without disturbing the morphological structure of Mn_3_O_4_ has significantly reduced the agglomeration and helped in electrons transfer facilitation at the interface of Mn_3_O_4_@rGO-10% nanocomposite modified GCE and electrolyte (Fig. 3C) therefore played a crucial role in the ORR. It can be observed through Mn_3_O_4_@rGO nanocomposites presented in Fig. 3B–D that Mn_3_O_4_ is sandwiched between various layers of rGO sheets. Therefore, blurry images of Mn_3_O_4_ nanograins were observed under rGO sheets, as circled in FESEM images. These sandwiched nanoparticles behave like spacers between the various rGO layers by letting the electrolyte diffuse into multiple nanostructure layers, resulting in an enhanced electrocatalytic activity. The nanocomposites Mn_3_O_4_@rGO-5% and Mn_3_O_4_@rGO-15% were also studied under FESEM. Mn_3_O_4_@rGO-10% (Fig. 3B) showed higher improved performance than other concentrations of GO for ORR, reflecting an optimum concentration of GO. The Mn_3_O_4_@rGO-5% nanocomposite containing 5 wt% of GO remains unsuccessful in preventing the agglomeration of Mn_3_O_4_ due to lower GO contents. This results in lower electrocatalytic performance for ORR. Moreover, a higher concentration of GO up to 15 wt% led to a higher number of transparent rGO sheets, which effectively decreased the concentration of the Mn_3_O_4_ catalyst. Hence, lower electrocatalytic performance was observed in the case of Mn_3_O_4_@rGO-15% for ORR (Fig. 3D).

TEM analysis was used to analyze the nanostructures of Mn_3_O_4_ and Mn_3_O_4_@rGo nanocomposites. Figure S1 clearly shows the agglomerated nanosized particle of unaided Mn_3_O_4_ nanograins after microwave-assisted hydrothermal synthesis, which is evident for metal oxide nanoparticles without any supporting matrix. Figure S1B–D distinctly shows the rGO (marked by arrows) supported Mn_3_O_4_ nanograins from bare Mn_3_O_4_ nanograins. The densely populated nanograins of Mn_3_O_4_ on the rGO surface in Figure S1(B) are due to the lowered concentration of GO (5 wt%) initially used for synthesizing nanocomposites. While figure S1(D) reveals that the less populated Mn_3_O_4_ nanograins distributed evenly on the rGO matrix are due to increased layers of rGO and confirms the highest concentration of GO used for the synthesis of Mn_3_O_4_@rGO-15% nanocomposites. At the same time, the optimized and balanced distribution of Mn_3_O_4_ nanograins was observed in figure S1(C) with 10 wt% of GO used for the synthesis of Mn_3_O_4_@rGO-10% nanocomposites. The TEM images have the same chemistry as explained in the FESEM images and the XRD analysis. Hence the TEM agreed well with the XRD and SEM analysis. The particle size of the Mn_3_O_4_@rGO nanocomposite was calculated using ImageJ software, and the average particle size was found to be 32 nm by considering 200 particles, which is in good agreement with the XRD results.

**Figure 4:**
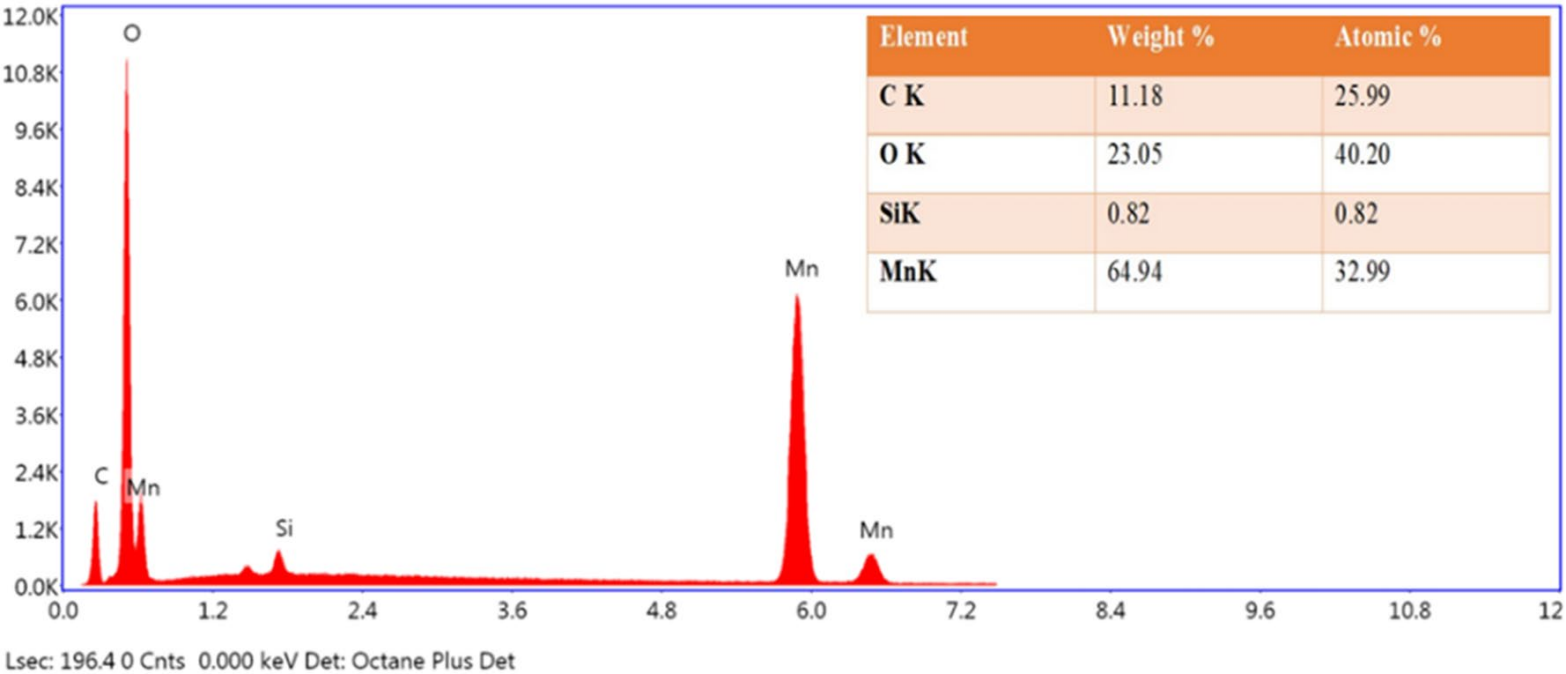
EDX analysis of Mn_3_O_4_@rGO-10% nanocomposite.

EDX was used to inspect the elemental distribution and purity of Mn_3_O_4_@rGO-10% nanocomposite, as shown in Fig. 4. The EDX spectrum showed prominent peaks related to Mn (64.94%), O (23.05%), and C (11.18%) without traces of other peaks, confirming the purity of the synthesized nanocomposite. The peak of Si (0.82%) was likely aroused from the substrate. The C peaks are related to the presence of rGO in the nanocomposite. The elemental distribution of unaided Mn_3_O_4_ nanograins was also tested using EDS, as shown in figure S2. The carbon-coated Cu grid was used for the EDS analysis, and the EDS spectrum shows the prominent peaks with atomic % related to the O and Mn with 50.66 and 34.32, respectively (inset figure S2). The wt% of Mn and O was also recorded and found as 61.4 and 26.4, respectively, without any extra peaks related to the involvement of impurities during the synthesis.

**Figure 5:**
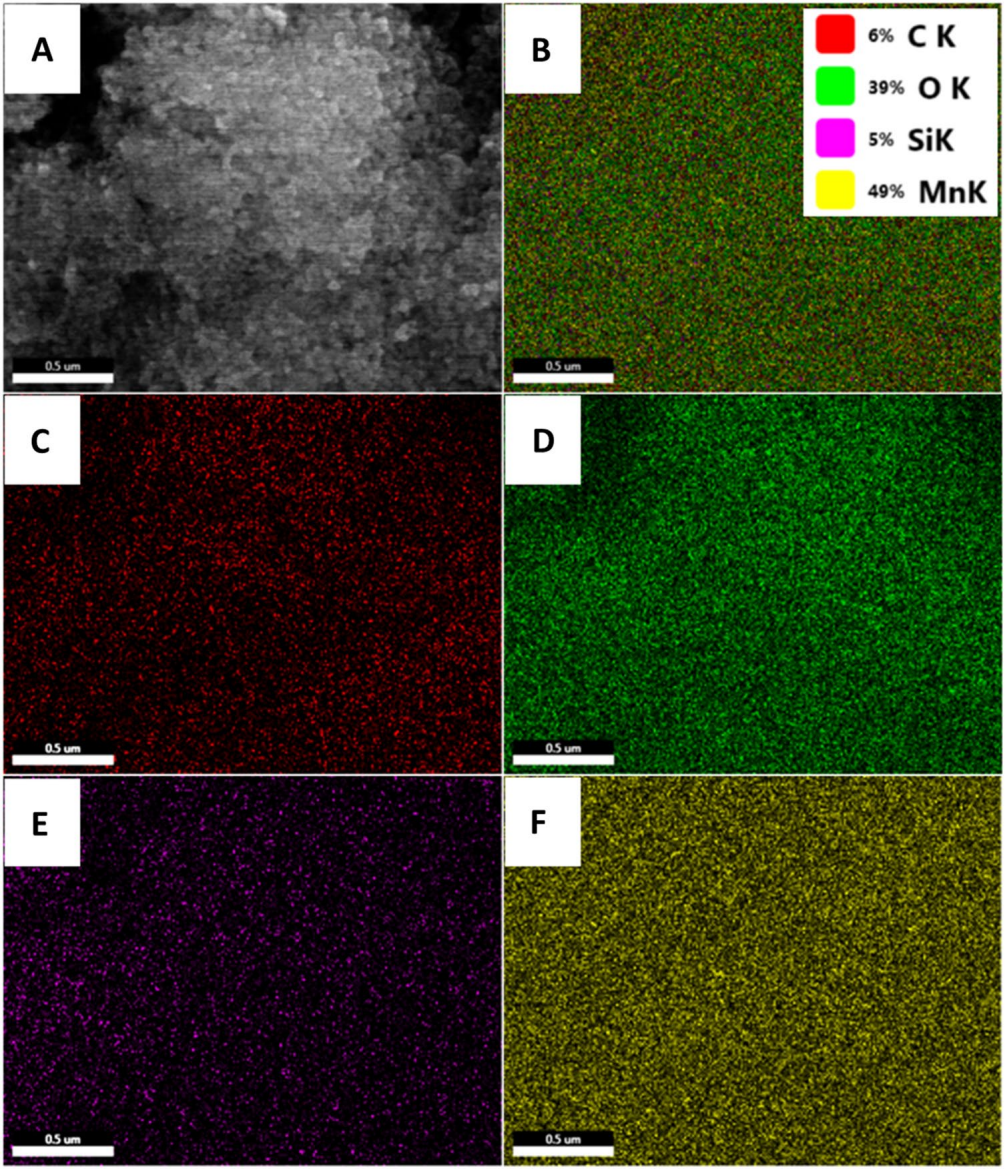
EDX elemental mapping images of Mn_3_O_4_@rGO-10% nanocomposite, (**A**) FESEM image, (**B**) Mix, (**C**) Carbon, (**D**) Oxygen, (**E**) Si wafer, and (**F**) Mn.

To validate the presence of each element, an element mapping analysis was performed, and the distribution of each component of the nanocomposite electrode materials is shown in Fig. 5. The combined elemental distribution ensures a homogeneous distribution of all elements in the nanocomposite of Mn_3_O_4_@rGO-10% (Fig. 5B). The mapping was derived from the FESEM image to analyze elemental distribution, as seen in Fig. 4A. The high density of yellow dots in Fig. 5F represents the densely distributed Mn nanoparticles on the carbon matrix (red color). In contrast, the green color represents the presence of oxygen in Mn_3_O_4_ nanoparticles (Fig. 5D). The purple color mapping image reflects the presence of the Si substrate used for analysis purposes (Fig. 5E). The presence of carbon as red color dots in Fig. 5C confirms the presence of rGO matric in the nanocomposite.

### Raman studies

Figure S3 (A &B) shows the Raman spectra of two fundamental vibrations ranging from 1100 to 1700 cm^−1^ for GO and rGO. The D band formed due to the breathing mode of j-point photons is visible at 1356.8 and 1351.12 cm^−1^ belonging to the A_1g_ symmetry of GO and rGO, respectively^51^. However, in figure S3(B), the first ordered scattered G vibration band belongs to E_2g_ phonons by sp^2^ carbon having peaks appearing at 1591.79 cm^−1^ belongs to GO and 1597.3 cm^-1^ for rGO^52^. Furthermore, the existence of the stretching C–C bond, which is typical for all sp^2^ carbon systems, also contributed to originating off the G vibration band in GO and rGO spectrums^53^. In the Raman spectrum, the disorder bands are represented by the D band, and the G band refers to tangential bands^52^. The 2D band is used to determine monolayer bilayer and multilayer graphene sheets and is very sensitive to stacking graphene layers. The shifted 2D band at 2694.93 cm^−1^ in figure S3B confirms a multilayer GO synthesis. Further, the shift in wavenumber for GO was also due to the presence of oxygen-containing functional groups, which helps to prevent the GO layer from stacking. Moreover, the 2D band for rGO appeared at a lower wavenumber (2686.32 cm^−1^) as compared to GO due to the reduction of GO into rGO and the presence of less number of oxygen functional groups, which causes the rGO layer to restack^51^.

The lower intensity ratio of the D to G band (0.87 < 1) shows the successful synthesis of GO, while after the microwave synthesis, the GO was reduced to rGO^54^, confirming the restoration of sp^2^ carbon, which resulted in higher D to G intensity ratio (I_D_/I_G_ > 1) and higher intensity of D band due to the removal of oxygen function moieties^55^. Figure S3(C) shows the Raman spectra of the Mn_3_O_4_@rGO-10% nanocomposite; the appearance of Raman modes in the range of 100–1000 cm^−1^ along with D, G, and 2D bands confirms the successful synthesis of nanocomposites. However, the high-intensity band at wavenumber 657.6 cm^−1^ refers to the A_1g_ mode due to oxygen ions motion inside MnO6 octahedra and is attributed to Jahn–Teller distortion. Besides this, the band with low-intensity peaks located at 372 and 319 cm^-1^ corresponds to the Mn–O bending modes and oxygen bridge species of asymmetric stretch (Mn–O–Mn), respectively^56^. Besides this, a shallow intense peak at 466.5 cm^−1^ appeared to be assigned to the E_g_ mode of Raman.

### UV–Vis analysis

**Figure 6:**
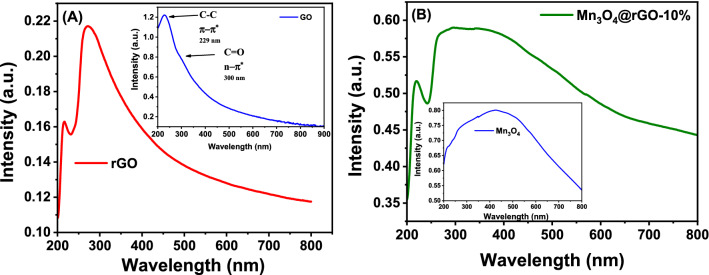
(**A**) UV–Vis spectra of rGO and GO (Inset), and (**B**) Mn_3_O_4_@rGO-10% nanocomposite with Mn_3_O_4_ as inset.

UV–Vis spectroscopy was used to examine the reduction of GO into rGO during a microwave hydrothermal process, as illustrated in Fig. 6A. The Soret band red shift was observed for rGO at 272 nm after the microwave hydrothermal reaction, indicating a more significant number of electron transfers from the rGO sheets. The inset in Fig. 6A shows a sharp GO peak at 229 nm for the π–π* transition of C–C bonds^51^. A fragile shoulder that appeared at 300 nm in the GO spectrum is associated with C=O bonds, which is consistent with the n-π* transition and supports the presence of the carbonyl group on the GO surface^57,58^. UV–Vis spectrum of Mn_3_O_4_ and rGO nanocomposite (Fig. 6B demonstrates the absorbance band of rGO at 220 nm, including the hump of Mn_3_O_4_ catered about 430 nm (Fig. 6B(inset), indicating the formation of Mn_3_O_4_@RGO nanocomposite. The inset in Fig. 6B shows that Mn_3_O_4_ exposes the lowest UV absorption regime and shows an absorption spectrum as a hump centered at around ~ 430 nm^59,60^.

### ATR-FTIR analysis

**Figure 7:**
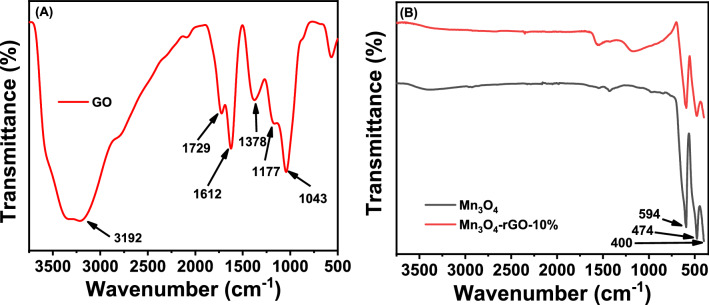
ATR-FTIR spectra of (**A**) GO and (**B**) Mn_3_O_4_ and Mn_3_O_4_@rGO-10% nanocomposite.

The chemical compositions of GO, Mn_3_O_4_, and Mn_3_O_4_@rGO-10% nanocomposites were investigated employing FTIR spectroscopy, as shown in Fig. 7. In Fig. 7A, due to the O–H stretching vibration of intercalated water, a wide band for GO emerged about 3192 cm^−1^^61^. The band at 1729 cm^−1^ is allocated to the C=O stretching mode of carboxylic acid and carbonyl moieties. In addition, the band at 1612 cm^−1^corresponds to the unoxidized graphitic domain^61^ or C=C bond of sp^2^ hybridized stretching vibration. The band at 1378 cm^−1^ was attributed to the stretching vibrations of C–OH^62,63^, and the shoulder bands appeared at 1176, and 1043 cm^−1^ are attributed to C–O epoxy starching and C–O alkoxy stretching, respectively^3,64–66^. Figure 7B shows characteristic bands between 400 to 600 cm^−1^ assigned to the stretching modes of Mn–O^67^. The band located at 594 cm^−1^ is attributed to the Mn–O stretching mode of tetrahedral sites, while the 474 cm^−1^ band is related to the distortion vibration of Mn–O at the octahedral sites. The band appearing near 400 cm^−1^ is likely attributed to the Mn vibrations in the Mn_3_O_4_ octahedral site^68^. The corresponding spectrum of Mn_3_O_4_@rGO-10% nanocomposite shows similar characteristic bands to those of Mn_3_O_4_ without extra absorption bands related to GO, indicating the reduction of GO to rGO in the nanocomposite was successful.

The electrical conductivity of all nanocomposites and Mn_3_O_4_ was measured by the two-probe method at room temperature by making pellets of the synthesized samples. Various data points were collected by selecting different positions on the pellets, and values are summarized in Table 1. The nanocomposites of Mn_3_O_4_@rGO have exhibited higher electrical conductivity for the increasing contents of rGO. Mn_3_O_4_ being an insulator has shown overflow and hence presented zero conductivity. On the other hand, the conductivity increased for Mn_3_O_4_@rGO-15% > Mn_3_O_4_@rGO-10% > Mn_3_O_4_@rGO-5%, summarized in Table 1. This increased conductivity was basically due to the higher wt% of GO used for the synthesis of nanocomposite, which resulted in rGO contents after reduction by microwave hydrothermal reactions. Basically, rGO is the source of electrical conductivity to the intercalated nanograins of Mn_3_O_4_.

**Table 1:** The electrical conductivity test of Mn_3_O_4,_ Mn_3_O_4_@rGO-5%, Mn_3_O_4_@rGO-10% > Mn_3_O_4_@rGO-15% materials.

S. No.	Material	GO wt% (%)	Conductivity (S m^−1^)
1	Mn_3_O_4_	0	1.3532 × 10^−6^
2	Mn_3_O_4_@rGO-5%	5	4.967 × 10^−3^
3	Mn_3_O_4_@rGO-10%	10	7.5331 × 10^−3^
4	Mn_3_O_4_@rGO-15%	15	4.8197 × 10^−2^

### Electrochemical behavior of Mn_3_O_4_@rGO-10% nanocomposite in [Fe(CN)_6_]^3-/4-^ and electrochemical impedance spectroscopy analysis

**Figure 8:**
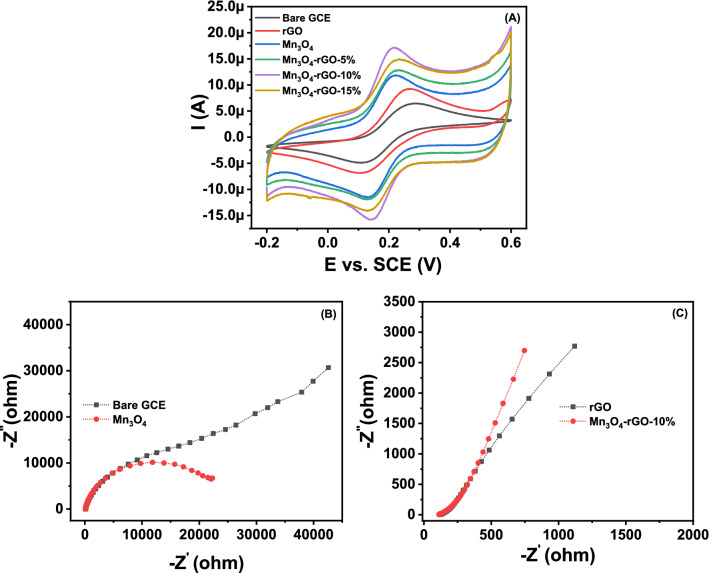
(**A**) Cyclic voltammetric curves taken for Bare GC, rGO, Mn_3_O_4_, and Mn_3_O_4_@RGO nanocomposite with different wt% of GO (5%, 10%, and 15%) modified GCE in 1 mM K_3_[Fe(CN)6] as analyte in 0.1 M KCl electrolyte at a scan rate of 50 mVs^−1^, (**B**) the EIS studies are taken into the exact solution for bare GCE and Mn_3_O_4_, (C) rGO and Mn_3_O_4_@rGO-10% nanocomposite modified GCE.

The redox behavior of [Fe(CN)_6_]^3-/4-^ was studied at the nanocomposite-modified GCE, including other controlled electrodes. This is a valuable technique for studying the kinetic barrier at the interface of a modified electrode and the electrolyte. The transfer of electrons at the electrode interface and the electrolyte solution occurs due to tunnelling from the defects present in the barrier or through the barrier^69,70^. Therefore, [Fe(CN)_6_]^3-/4-^ redox couple was selected as a standard marker to study the change in behavior of different synthesized cathode materials, as it is a frequently used diagnostic tool for elucidating the mechanism of modified electrodes. Considering the redox chemistry viewpoint, using ferricyanide or ferrocyanide as a redox couple is a one-electron transfer phenomenon at room temperate. As can be seen through Fig. 8A in the forward scan of CV at bare GCE/Modified GCE surface, the ferrocyanide is oxidized and converted into ferricyanide by donating one electron. During the cathodic sweep or reverse scan, ferricyanide reduction started and reverted to ferrocyanide by accepting one electron. This continuous process shows that the ferricyanide/ ferrocyanide redox reaction is a single electron transfer process^71,72^.

Figure 8A shows the results from various electrode materials using cyclic voltammetry in 0.1 m KCl as electrolyte solution and 1 mM ferricyanide as analyte at a scan rate of 50 mV/s.

The bare GC electrodes showed a well-defined CV with a diffusion-limited redox process. After modifying the GC electrode surface, the redox peak current increase gradually on the order of Mn_3_O_4_@rGO-10% > Mn_3_O_4_@rGO-15% > Mn_3_O_4_@rGO-5% > Mn_3_O_4_ > rGO due to the facilitation and tunnelling of a more significant number of electrons through the defects or the barrier. This is due to the conductivity of rGO (in the composite) and the contribution of evenly distributed Mn_3_O_4_ nanograin electroactive surface area on rGO sheets, which facilitate more significant and faster electron transfer numbers. Noticeably, the peak potential separation (ΔE) decreased with the increase in the redox current for Mn_3_O_4_@rGO-10%, compared to other controlled electrodes. The decrease in the anodic and cathodic peak currents by Mn_3_O_4_@rGO-15% nanocomposite was also observed compared with Mn_3_O_4_@rGO-10% nanocomposite. The increasing number of rGO sheets is predicted to reduce the density of the Mn_3_O_4_ nanoparticles, which coincides with the FESEM picture in Fig. 3.

The EIS studies were also carried out in line with the cyclic voltammograms in ferricyanide containing KCl electrolyte at the frequency ranges from 0.01 to 100 000 Hz to investigate the charge transfer facilitation at the interface of the modified GCE and the electrolyte (Fig. 8B and C)). The Nyquist diagram shows the real (**Z**^**’’**^) versus imaginary (**Z**^**’**^) components of the complex impedance with a semicircle showing the charge transfer resistance at higher frequencies and the straight line corresponding to the diffusion-limited process in the low-frequency region^73^. The GC electrode shows a higher *R*_*ct*_ value of 30 K Ω, compared with the Mn_3_O_4_ modified GC electrode (*R*_*ct*_ = 25.875 K ohm) by following the trajectory of the semicircle at high frequencies. This confirms the resistance at the electrode and electrolyte interface, which hinders the flow of charges. The Mn_3_O_4_@rGO-10% and rGO-modified GC electrode was investigated. No semicircle was formed for both the modified electrodes, showing the lowest charge transfer resistance and faster electron transfer. The straight light adjoining the semicircle for all electrodes shows the Warburg impedance of all electrodes at higher frequencies. The Warburg impedance with an angle slightly more elevated than 45° represents that the electrode doesn’t behave like a capacitive material^74^.

### Electrochemical ORR studies

**Figure 9:**
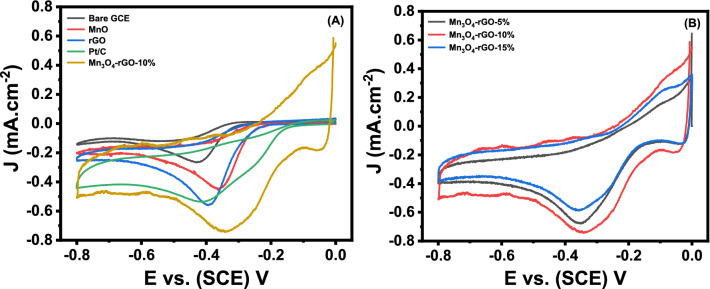
Cyclic voltammetry curves of (**A**) Bare GCE, Mn_3_O_4_, rGO, Pt/C, and Mn_3_O_4_@rGO-10% in O_2_ saturated 0.5 M KOH at a scan rate of 50 mV/s, (**B**) the CV comparison of Mn_3_O_4_@rGO nanocomposite with other GO wt% (5%,10%, and 15%) in O_2_ saturated KOH under the same conditions.

The electrochemical behavior of modified GCE with all the synthesized electrode material was examined for ORR as fuel cell application in an O_2_-saturated 0.5 M KOH (Fig. 9A). The cyclic voltammetric approach was applied at a scan rate of 50 mV/s for O_2_ reduction within the potential range of 0.0 to − 0.8 V. All controlled electrodes were also employed in the ORR activity. The bare GCE contributed a very low current density peak of 0.266 mA/cm^2^ when compared with Mn_3_O_4_ modified GCE (− 0.448 mA/cm^2^) with a low overpotential of − 0.358 V. The rGO being a conducting 2D material with extraordinary electrical and electronic properties, has performed better than the Mn_3_O_4_ modified and bare GCE with a current density of -0.562 mA/cm^2^ but with a slightly higher overpotential value of -0.395 V due to the restacked wrinkled sheets after reduction of GO into rGO. The unaided Mn_3_O_4_ nanoparticle has shown poor electrocatalytic performance due to the agglomerated nature of metal oxides when compared with Mn_3_O_4_@rGO nanocomposites. The electrocatalytic activity was dramatically increased for ORR when nanocomposite-modified GCE was employed in O_2_ saturated KOH electrolyte. The Mn_3_O_4_ nanoparticles and the high conduction rGO in the nanocomposites have contributed to higher electrocatalytic performance with a current density of − 0.738 mA/cm^2^ shown by Mn_3_O_4_@rGO-10% nanocomposite. It was due to the synergistic effect, which helped shift potential towards a positive side at the value of − 0.345 V and a well-defined peak of ORR^75^. It is well known that the removal of oxygen functional groups causes the layers of rGO to stack after reduction by the hydrothermal process. Therefore, as alone do not perform very well.

Similarly, metal oxide nanoparticles also exist in aggregated form after synthesis, due to which their participation in electrochemical reactions becomes limited^76^. Using rGO as conducting platform for metal oxide nanoparticles can prevent agglomeration and aggregation. In return, the metal oxide nanoparticle prevents the rGO layers from stacking, and behaves as spacers between the layers of rGO. This synergistic effect of metal oxide nanoparticles and rGO can tremendously boost the performance of electrocatalysts for ORR^77^. Mn_3_O_4_ and rGO nanocomposite was synthesized by considering the same phenomenon. This helps in increasing the overall geomaterial surface area of Mn_3_O_4_ by preventing them from agglomeration and, at the same time, spacing between the rGO layers due to the intercalation of Mn_3_O_4_ nanoparticles. This helps the electrolyte to diffuse into the various layer of rGO by increasing the electrochemically active surface area and boosts the ORR performance of the cathode material. The commercially available Pt/C catalyst was also exploited for the ORR performance as it is the commonly used electrocatalyst in fuel cells studies, which shows the current density of − 0.536 mA/cm^2^ at a higher overpotential of − 0.414 V. Besides, as can be seen through Fig. 9B that Mn_3_O_4_@rGO-5% and Mn_3_O_4_-rGO-15% nanocomposite was also studied by dipping into the O_2_ saturated KOH as an electrolyte solution. It was noticed that Mn_3_O_4_@rGO nanocomposite-modified GCE with 5 and 15 wt% of GO contents showed lower electrocatalyst performance as compared to Mn_3_O_4_@rGO-10% nanocomposite, reflecting the optimized contents of GO wt% for ORR studies. The nanocomposite with 5 wt% of GO contents has shown poor performance due to the availability of less active catalytic sites. Further, it was also observed that increasing the content of GO up to 15 wt% also hinders the performance of the electrode material due to an increase in the number of layers which increases the thickness of the diffusion layer^3^. The higher GO contents are responsible for lower Mn_3_O_4_ nanograins density, influencing the electrochemically active surface area. This hinders the electron transfer at the interface of electrode and electrolyte, which is also confirmed by the EIS and CV results carried out in ferricyanide redox couple contained in KCl as electrolytes solution.

Similar work has been done previously by using manganese oxide and reduced graphene oxide nanocomposite as the primary electrode material for ORR in fuel cells and other energy conversion devices. Table 2 here summarizes the reduction of O_2_ with various parameters and compares the analytical performance of the present nanocomposite with the reported work. The current work displayed satisfactory performance compared with the reported work and stands for its rapid and time-saving one-pot microwave-assisted hydrothermal synthesis for developing cathode material.

**Table 2:** A comparison of reported work based on manganese oxide-reduced graphene oxide nanocomposite for ORR.

S.No	Material	Synthesis method	Analysis method	Potential	Current density	Reference
1	MnO_2_/rGO MnO_2_/PEDOT/rGO	electrochemical method	Cyclic voltammetry	− 0.4 V and − 0.3 V	1 mA cm^−1^ and 1.75 mA cm^−1^	^78^
2	rGO/MnO_2_/Ag	electrochemical deposition	Cyclic voltammetry	~ 0.9 V	0.98 mA cm^−1^	^79^
3	MnO_2_/RGO composites	polymer-assisted chemical reduction method	Cyclic voltammetry	~ − 0.05 V	− 0.004 mA cm^−1^	^80^
4	Mn_3_O_4_/rGO composites	reflux method	Cyclic voltammetry	− 0.299 V	− 4.0 mA cm^−2^	^81^
5	Mn_3_O_4_-rGO/C	in situ generation	Cyclic voltammetry	− 0.2 V	− 4.1 mA cm^−2^	^82^
6	Mn_3_O_4_/ rGO composite	hydrothermal process	Cyclic voltammetry	− 0.23 V	0.38 mA cm^−2^	^83^
7	RGO–MnO_2_	–	Linear sweep voltammetry	− 0.3 V	− 0.2 mA cm^−2^	^30^
8	Mn_3_O_4_@rGO	Microwave Hydrothermal process	Cyclic voltammetry	− 0.345 V	− 0.738 mA/cm^2^	This work

**Figure 10:**
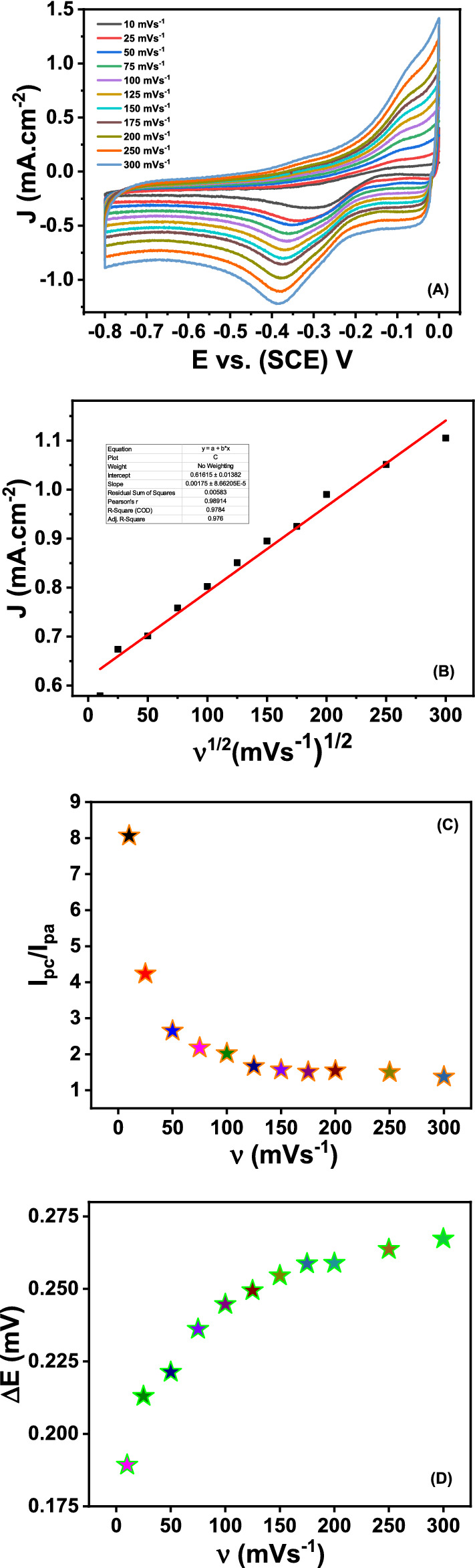
(**A**) Cyclic voltammetry taken at Mn_3_O_4_@rGO-10% modified GCE at different scan rates (10–300 mVs^−1^) in O_2_ saturated 0.5 M KOH, (**B**) the calibration plot of the square root of scan rate *vs* cathodic current, (**C**) plot of (i_pc_/i_pa_) *vs.* scan rate, (**D**) plot of DE_p_ *vs.* scan rate.

The cyclic voltammograms were taken at different scan rates for ORR using Mn_3_O_4_@rGO-10% nanocomposite modified GCE in 0.5 M KOH electrolyte solution (Fig. 10A). There is an obvious increase in cathodic peak current that can be seen with increased scan rate. The peak current for ORR corresponding to the square root of scan rates has shown a linear relationship in Fig. 10B. The increase in the reduction current of O_2_ by increasing the scan rate from 10 to 300 mV/s at nanocomposite-modified electrodes suggested that it is a diffusion-controlled process^2^. The scan rate studies reveal that the reduction peak current increases with the increase in the scan rate. Meanwhile, it was observed that the oxidation peaks also appear with very weak and broad peaks. From the CV measurements, it is confirmed that the ratio of reduction peak current and oxidation peaks current (i_pc/_i_pa_) is decreasing with the increase in scan rate (Fig. 10C). The peak’s potential difference ΔE_p_ through the cyclic voltammogram is increasing with the increase in scan rate (Fig. 10D). These observations collectively confirm that the ORR process is quasi-reversible and suggest two electrons transfer at the surface of the modified electrode, which led to the reduction of O_2_ into the formation of OH_2_^-^ in alkaline media as expressed in the following Eq. 1^84^. As expressed in the chemical reaction in Eq. 2, the peroxide ions (${\text{HO}}_{{2^{ - } }}$) are either obtained by further reduction (Eq. 2) or by the catalytic breakdown of peroxide ions (Eq. 3)^85^.

1 $${O_2+H_2 O +2e^-}\rightarrow{HO_{2^{-}}+OH^{-}}$$

2 $$HO_{2^{-}}+H_{2}O+2e^{-}=3OH^{-}$$

3 $$2HO_{2^{-}}\rightarrow O_{2} + 2OH^{-}$$

The electron is transported from different rGO layers when it reaches Mn ions. This would create high positive charges on the surface of the Mn_3_O_4_@rGO-10% nanocomposite. These electrons cause an unbalanced charge distribution, which greatly prefers to adsorb the O_2_ and increases the rate of the gas diffusion process. Since Mn_3_O_4_ nanoparticles firmly adhere to a highly conducting rGO surface, this leads to efficient ionic and larger electron transportation^85–87^.

**Figure 11:**
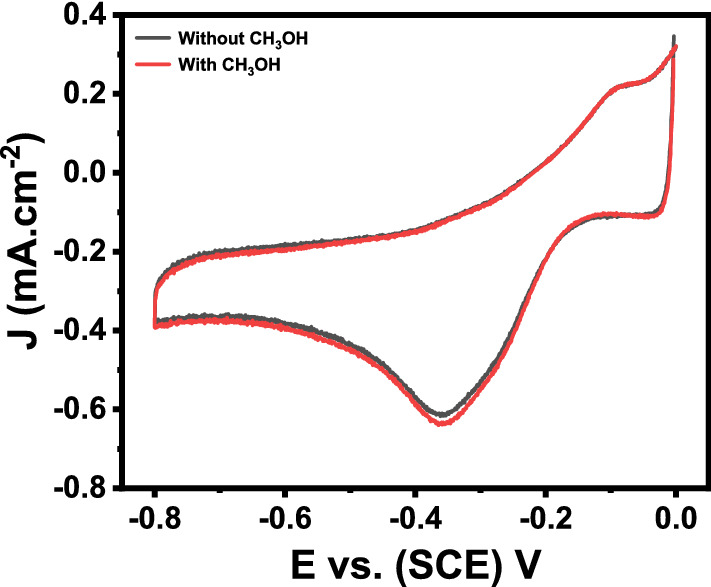
Cyclic voltammograms were taken at Mn_3_O_4_@rGO-10% nanocomposite modified GCE in 0.5 M O_2_ saturated KOH at a scan rate of 50 mVs^−1^ in the presence and absence of 0.5 M CH_3_OH.

Methanol tolerance is the key factor to discuss in fuel cell applications, especially in ORR. Because using methanol as a fuel in DMFC, the methanol gas can permeate to the cathode chamber and poison the catalyst due to cross-over effects which significantly reduces the performance of the catalyst. The methanol tolerance was evaluated using Mn_3_O_4_@rGO-10% nanocomposite-modified GCE electrocatalyst in the presence of a higher concentration (0.5 M) of CH_3_OH in O_2_-saturated 0.5 M KOH (Fig. 11). The comparison of the cyclic voltammograms is shown in Fig. 11 in the presence and absence of CH_3_OH, which shows that the CV remains unchanged even in the presence of a higher concentration. These results show that the electrocatalyst is highly selective towards ORR and not susceptible to any foreign molecules.

**Figure 12:**
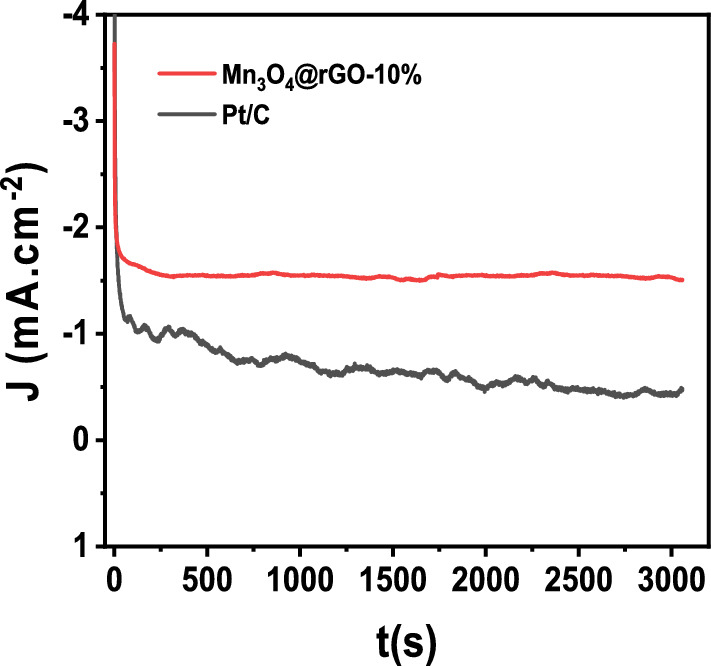
For stability analysis, the Chronoamperometric (i-t) curves of Mn_3_O_4_@rGO-10% nanocomposite and Pt/C catalyst-modified GCE in O_2_ saturated 0.5 M KOH solution.

The stability of Mn_3_O_4_@rGO-10% nanocomposite-modified GCE was studied by recording a long-term chronoamperometric (CA) current time (*i-t)* curve in an O_2_-saturated KOH solution (Fig. 12). The *i-t* curve was recorded for Mn_3_O_4_@rGO-10% and Pt/C at a potential recorded from the reduction peaks of the cyclic voltammetric curve to obtain the stability curve. It was noticed that even after over 3000 s, the Mn_3_O_4_@rGO-10% nanocomposite curve remained unchanged and maintained high current retention of 98% without any susception by the surrounding environment. On the other hand, the CA obtained for Pt/C has loosened its current retention to 58% after 3000 s, which was a 29-time higher decrease compared to Mn_3_O_4_@rGO-10% nanocomposite. These results revealed that Mn_3_O_4_@rGO-10% is a highly durable catalyst for ORR.

## Conclusions

In summary, the composite of Mn_3_O_4_@rGO nanocomposites was produced by the microwave-assisted hydrothermal method, which is a simple, fast, and scalable synthesis method. Mn_3_O_4_ and rGO, when combined as hybrid materials, exhibited surprising ORR activities in an alkaline medium. The synthesized nanograins of Mn_3_O_4_ were evenly distributed on the rGO matrix. The functional group present on GO matrix prevented the Mn_3_O_4_ nanograin from aggregation, and intercalation of Mn_3_O_4_ nanograins further helped avoiding the rGO sheets from restacking behaving as spacers and hence resulted in higher ORR activity. The Mn_3_O_4_@rGO-10% nanocomposite has shown the highest ORR activity with a current density of − 0.738 mA/cm^2^ at the shifted potential of − 0.345 V. The tolerance against a higher concentration of methanol was tested by CV, which was present in O_2_-saturated KOH; it was noticed from CV curves that Mn_3_O_4_@rGO-10% nanocomposite exhibited outstanding performance, and it was confirmed that the methanol crossover would not hamper the fuel cell performance. The stability test was conducted using a chronoamperometric technique for 3000 s. It was found that Mn_3_O_4_@rGO nanocomposites showed far exceeding stability and durability when compared with commercially available Pt/C catalysts. According to the findings, the microwave-assisted hydrothermal technique of nanocomposite synthesis is an efficient method of producing advanced electrode materials for numerous energy related fields.

## Supplementary Information

Supplementary Information.

## Data Availability

http://www.crystallography.net/cod/1514121.html.
