# Supplementary material for: Microwave assisted synthesis of Mn3O4 nanograins intercalated into reduced graphene oxide layers as cathode material for alternative clean power generation energy device
Source: Sci Rep. 2022 Nov 9;12:19043. doi: 10.1038/s41598-022-23622-x (PMC9646735; doi:10.1038/s41598-022-23622-x)
Supplement: Supplementary file 1 — Supplementary Information. [file 41598_2022_23622_MOESM1_ESM.docx]

**Supplementary Information**

**Microwave assisted synthesis of Mn_3_O_4_ nanograins intercalated into reduced graphene oxide layers as cathode material for alternative clean power generation energy device**

Mehmood Shahid^1*^, Thilina Rajeendre Katugampalage^1^, Mohammad Khalid^2^, Waqar Ahmed^3^, Chariya Kaewsaneha^1*^, Paiboon Sreearunothai^1^, Pakorn Opaprakasit^1*^

^1^School of Integrated Science and Innovation, Sirindhorn International Institute of Technology (SIIT),

Thammasat University, Pathum Thani 12121, Thailand

^2^Graphene and Advanced 2D Materials Research Group (GAMRG), School of Engineering and Technology, Sunway University, No. 5, Jalan Universiti, Bandar Sunway, Subang Jaya, Selangor 47500, Malaysia

^3^Malaysia – Japan International Institute of Technology (MJIIT), Universiti Teknologi Malaysia, Jalan Sultan Yahya Petra, 54100 Kuala Lumpur, Malaysia

Corresponding authors emails: shahid.mehmoodawan1@gmail.com

chariya@siit.tu.ac.th

pakorn@siit.tu.ac.th


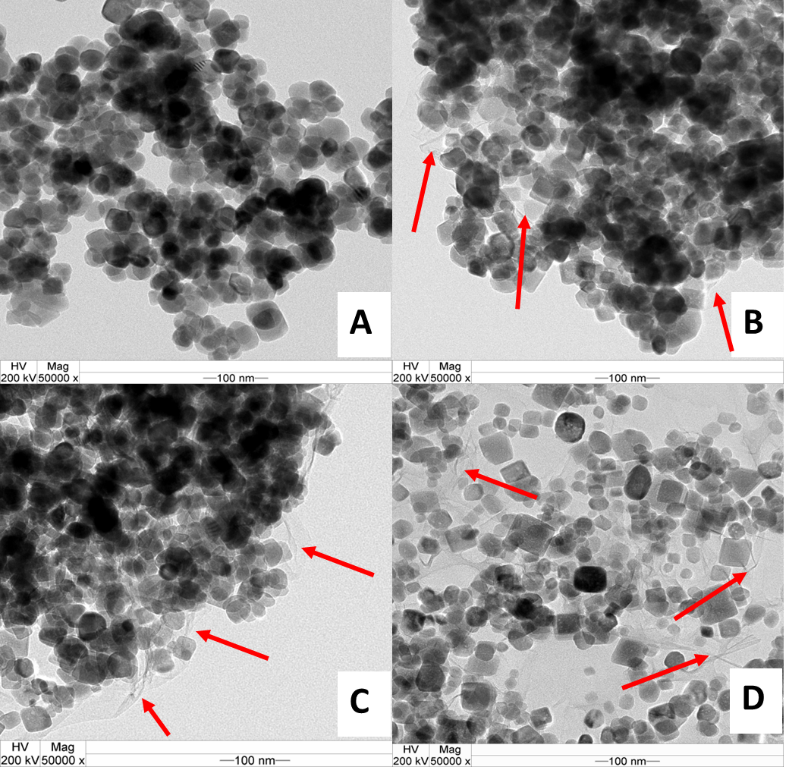


**Figure S1.** TEM images of (A) Mn_3_O_4_, (B) Mn_3_O_4_@rGO-5%, (C) Mn_3_O_4_@rGO-10%, and (D) Mn_3_O_4_@rGO-15%.


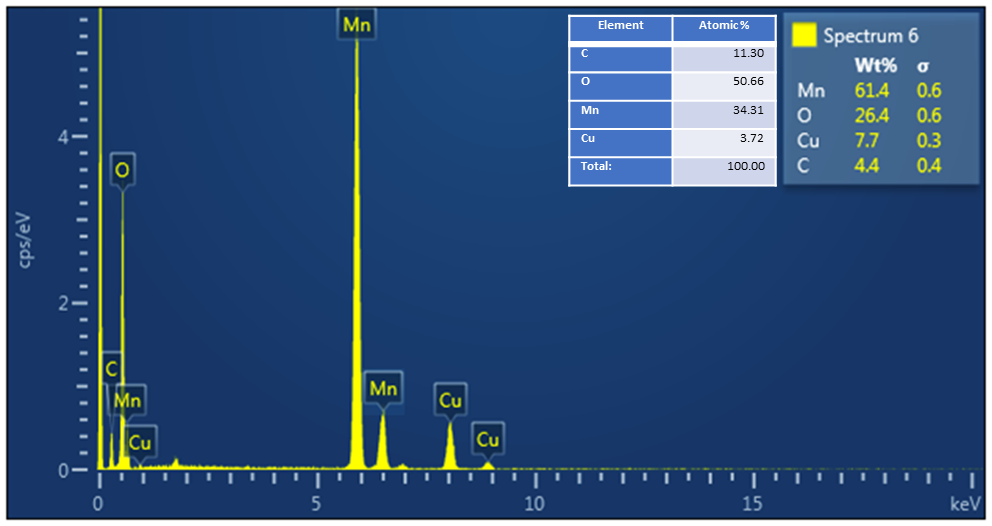


**Figure S2.** EDS analysis of unaided Mn_3_O_4_ nanograins

**Figure S3.** raman spectra of (A) GO, (B) rGO, and (C) Mn_3_O_4_@rGO-10% nanocomposite.
